# Development of an *in vitro* drug screening assay using *Schistosoma haematobium* schistosomula

**DOI:** 10.1186/1756-3305-5-165

**Published:** 2012-08-09

**Authors:** Monika Marxer, Katrin Ingram, Jennifer Keiser

**Affiliations:** 1 Department of Medical Parasitology and Infection Biology, Swiss Tropical and Public Health Institute, P.O. Box, CH–4002, Basel, Switzerland; 2 University of Basel, CH–4003, Basel, Switzerland

**Keywords:** In vitro, Chemotherapy, Schistosoma haematobium, Newly transformed schistosomula, Alamar Blue®, Cercarial rhythm, Percoll®, *Bulinus truncatus*, *Biomphalaria glabrata*

## Abstract

**Background:**

The development of novel antischistosomal drugs is crucial, as currently no vaccine and only a single drug is available for the treatment of schistosomiasis. Fast and accurate *in vitro* assays are urgently needed to identify new drug candidates and research efforts should include *Schistosoma haematobium*. The aim of the present study was to develop a *S. haematobium* drug sensitivity assay based on newly transformed schistosomula (NTS).

**Methods:**

We first undertook comparative studies on the cercarial emergence rhythms of the intermediate host snails *Biomphalaria glabrata* (*S. mansoni*) and *Bulinus truncatus* (*S. haematobium*). Two transformation methods as well as three purification methods were studied on *S. haematobium* cercariae in order to produce a large number of viable and clean NTS. Known antischistosomal drugs were tested in the established NTS assay *in vitro*. Drug effects were evaluated either microscopically or fluorometrically, using a resazurin based viability marker. Microscopically obtained IC_50_ values were compared with results obtained for *S. mansoni*.

**Results:**

A circadian rhythm existed in both snail species. Infected *B. truncatus* snails shed less cercariae than *B. glabrata* during the testing period. The highest transformation rate (69%) of *S. haematobium* cercariae into NTS was obtained with the vortex transformation (mechanical input) and the highest purification factor was observed using Percoll®. The fluorimetric readout based on resazurin was very precise in detecting dead or/and severely damaged schistosomula.

**Conclusions:**

With the use of viability markers such as resazurin, drug screening assays using *S. haematobium* NTS can be efficiently performed. However, drugs acting on the morphology and motility of *S. haematobium* NTS, such as metrifonate are missed. Drug sensitivity assays with NTS of both species, *S. haematobium* and *S. mansoni*, showed very similar results using known antischistosomal drugs. The *S. mansoni* NTS assay might be more suitable as primary screen in drug discovery efforts, which ultimately aim for a broad-spectrum antischistosomal drug as a larger number of *S. mansoni* NTS can be generated.

## Background

Schistosomiasis remains one of the most prevalent helminthic infections in the world. Human schistosomiasis is caused by three main species including *Schistosoma haematobium* and *Schistosoma mansoni.* Schistosomes have a complex life cycle with snails serving as the intermediary host between the mammalian hosts. Approximately 800 million people are at risk of schistosomiasis in 74 developing countries, with an estimated 200 million people currently infected. Schistosomiasis is a neglected tropical disease, however, just ranks below malaria in terms of its public health importance [1-4].

Control of schistosomiasis relies on preventive chemotherapy programs targeting the entire at risk population [5]. Up to now, praziquantel has been the drug of choice [6-8]. The drug is safe, has a broad therapeutic profile, and is cheap. However, these advantages have impeded the advances in the development of new drug candidates and vaccines [8,9]. In addition, praziquantel is not a perfect drug. It has a poor efficacy against immature worms, leading to a decreased ability of praziquantel to completely clear infections [8,9]. In addition, with the threat of drug resistance that accompanies preventive chemotherapy programs it is imperative to develop a novel antischistosomal drug. This drug should have a broad spectrum of trematocidal activity, similar to praziquantel. Given the known biological differences between *S. mansoni* and *S. haematobium,* drug screening should include testing against *S. haematobium*.

In the past years efforts have been made to improve drug sensitivity *in vitro* assays with *S. mansoni* enabling the testing of a larger number of compounds and improved readouts such as those with Alamar Blue® (a resazurin based viability marker) [10]. However, to our knowledge drug screening assays with *S. haematobium* continue to rely mainly on the adult worm.

The aim of the present work was to develop and validate an *in vitro* drug screening assay for *S. haematobium* newly transformed schistosomula (NTS). In a first step the cercarial emergence rhythms of the intermediate hosts of *S. mansoni* and *S. haematobiumBiomphalaria glabrata* and *Bulinus truncatus* were studied. Next, two artificial transformation methods for the production of the schistosomula were compared. In addition, the best purification method and optimal culture conditions were established by testing three different methods and several different media [11-14]. Finally, an assay based on the viability marker resazurin, which is the active component of Alamar Blue® and a redox indicator of enzyme activity, using selected compounds of known antischistosomal activity such as praziquantel, mefloquine, artesunate, metrifonate, and oxamniquine was established and the results compared to a standard motility assay using microscopical readout. Results were compared with drug activity observed microscopically against *S. mansoni* NTS.

## Methods

### Drugs and media

Praziquantel (Sigma Aldrich), mefloquine (kindly provided by Mepha Pharma AG, Switzerland), artesunate (kindly provided by Mepha Pharma AG, Switzerland), metrifonate (kindly provided by Bayer Animal Health, Germany), and oxamniquine (kindly provided by Q. Bickle, London School of Hygiene and Tropical Medicine) were used as antischistosomal compounds. Drugs were dissolved in dimethylsulphoxide (DMSO) (Sigma Aldrich) to obtain drug stock solutions of 10 mg/ml and then diluted into culture media. A resazurin solution was prepared by dissolving 12.5 mg resazurin (Sigma Aldrich) in 100 ml of 1x PBS. Different media such as Basch Medium 169 (prepared in our laboratories), Dulbecco’s Modified Eagle’s Medium (DMEM) (Gibco) and Medium 199 (Invitrogen, Carlsbad, CA) were tested and compared to obtain optimal culture conditions for the *S. haematobium* NTS. All test media were supplemented with 5% heat-inactivated fetal calf serum (FCS) based on the culture media protocol of Bash 1981 [12] and 200 U/ml penicillin and 200 μg/ml streptomycin (Sigma Aldrich).

### Snails and schistosomes

*Bulinus truncatus* infected with *S. haematobium* were obtained from BEI Resources, NIAID (NIH: *Schistosoma haematobium* exposed *Bulinus truncatus* subsp. *truncatus*, NR-21965). *S. mansoni* infected *B. glabrata* are maintained at the Swiss Tropical and Public Health Institute in Basel. The snails had been individually infected with either 10 miracidiae per snail (*B. truncatus*) or 8 miracidiae per snail (*B. glabrata*). Snails were kept in tanks with dechlorinated tap water in a humid room simulating a 12 hour day and night cycle. First shedding of cercariae occurred 4–6 weeks post infection. Both snail species were then collected in the morning (Size of snails: 6 – 11 mm, number of collected snails: approximately 70–90 of each species) and placed individually into 24 or 48 well plates (1 ml dechlorinated tap water/ well). Each well plate was placed under a direct 2000 lux lamp for at least 5 hours. The cercarial suspension of each *B. glabrata*/*B. truncatus* was collected and used for the mechanical and chemical transformation. For the cercarial emergence rhythm studies, *B. glabrata*/*B. truncatus* were collected (n = 90) in the morning at 8 am and put, as described above, in dechlorinated water filled well plates (1 ml/well) until 3 pm. During this period the cercariae suspension of each snail was collected hourly and counted for each snail under a light microscope. The hourly collected cercariae suspension was replaced with dechlorinated tap water.

### Vortex transformation

The vortex transformation was performed based on a slightly adapted protocol of Ramalho-Pinto [11]. Briefly, a cercarial suspension was placed on ice for 30–40 minutes in order to reduce parasite motility. Tubes were then centrifuged for 3 minutes at 3000 rpm. The cercarial pellet was resuspended in 2 ml cold Hanks` Balanced Salt Solution (HBSS) containing 2% amphotericin B (Sigma Aldrich). The suspension was vigorously mixed through a pipette followed by 4 minutes vortexing in order to induce tail shedding.

This step was repeated after an incubation of the mixed tail-schistosomula suspension for 20 minutes at 37°C. The transformation rate was calculated by counting the total number of cercariae in the HBSS suspension before transformation, and placing them in relation to the total number of schistosomula obtained after purification. The transformation rate was calculated for five identically performed mechanical transformations.

### Glucose induced transformation

The chemical transformation was carried out as described previously [11]. The cercariae suspension was cooled on ice for 30 minutes to reduce parasite motility. After 2 minutes of centrifuging at 2000 rpm, the cercariae suspension was resuspended in 4 ml of 5% glucose and incubated for 10 minutes in a 30°C waterbath. The tails were removed from the bodies using the ice purification method (see next paragraph). The transformation rate was calculated as described above and was calculated for five identically performed chemical transformations.

### Purification and culture of schistosomula

The separation of the bodies from the tails by centrifugation on a 70% Percoll® gradient (polyvinylpyrolidone-coated colloidal silica particles) was based on the method of Lazdins *et al*. [13]. 71.7 ml of Percoll® (Sigma Aldrich, starting density 1.13 g/ml) was diluted directly in 10 ml 1.5 M NaCl and filled up to 100 ml with distilled H_2_O to obtain a final working solution. 10 ml of the working Percoll® solution was layered on the bottom of a 50 ml Falcon tube. The gradient was topped by carefully adding 5 ml of the transformed schistosomula suspension. The tube was then centrifuged for 10 minutes at 2000 rpm and 5°C. After centrifugation, 5 ml of the sample was collected from the bottom of the tube after perforation using a heated 16-gauge needle. The collected fraction was then diluted in 7 ml of Medium 199 and centrifuged for another 3 minutes at 3000 rpm. The schistosomula pellet was resuspended in 1 ml of fresh, warm supplemented Medium 199.

Another purification method of the schistosomula was based on a simple and easy swirling technique according to F. Lewis *et al*. [14]. The schistosomula suspension was placed on a Petri dish and sufficient warm Medium 199 added so that the bottom of the Petri dish was completely covered. By swirling the dish gently, all schistosomula accumulated in the center and could be transferred into Falcon tubes. The swirling and collecting step was repeated until schistosomules were no longer present in the center of the dish (approximately 4–5 times).

In addition, the ice method was tested for purification. 7 ml of cold HBSS was added to the schistosomula suspension and cooled on ice for 7 minutes. The supernatant was decanted, and the pellet resuspended again in 7 ml of cold HBSS. This step was repeated three times. The pellet that contained the recovered schistosomula was then resuspended in preheated (37°C) supplemented Medium 199.

After the experiment the number of heads and tails were counted using a sample of 50 μl. The ratio was expressed as purification factor. Every method was performed three times identically and the mean purification factor was calculated.

After successfully transforming cercariae into schistosomula and purifying them, the NTS were incubated for at least 12 hours in supplemented Medium 199 at an atmosphere of 37°C with 5% CO_2_ until usage to assure complete transformation [10].

Different media, namely supplemented Basch Medium 169, Dulbecco’s Modified Eagle’s Medium and Medium 199 were tested and compared to obtain optimal culture conditions for the *S. haematobium* NTS. Viability of NTS (150–200 per media) was assessed based on their morphology and motility using a viability scale ranging from 3 (normal activity, no morphological changes) to 0 (dead), as described previously using increments of 0.5 [15].

### *In vitro S. haematobium* drug assay with microscopical or fluorimetric readout

NTS were obtained by mechanical transformation of *S. haematobium* cercariae as described above. The schistosomula suspension was adjusted to a concentration of 2 NTS per μL with supplemented Medium 199. A 3-fold serial dilution was next performed vertically down a flat bottom 96-well plate, to obtain final drug concentrations of 1.1, 3.3, 10, 30, 90 μg/ml after adding 50 μl of the adjusted NTS suspension yielding a final volume of 250 μl per well [16]. Each drug concentration was tested in duplicate and performed at least three times. Live and dead schistosomula (treated with 70% ethanol) served as a positive and negative control.

For the microscopical readout, assays were evaluated under an inverted bright-field light microscope (Carl Zeiss AG, 8 x 10 magnification) 24, 48 and 72 hours post drug exposure.

NTS were evaluated using the viability scale as described above. IC_50_ values were calculated using CompuSyn software (Version 3.0.1, 2007; ComboSyn, Inc).

Furthermore, drug effects were determined with the help of resazurin, a fluorimetric marker for cell viability [17]. Assays were conducted as described above with the exception that 48 hours post drug exposure 20 μl of the prepared resazurin solution was added to each well. Background fluorescence and absorbance of the drug containing medium were determined for each drug dilution. Wells without drug served as controls. After another 24 hours of incubation, fluorescence development was determined (after a total drug incubation time of 72 hours). Fluorescence was measured using a Spectramax M2 plate reader (Molecular Devices) at 530 nm excitation wavelength and 590 nm emission wavelength. The IC_50_ of each drug was calculated based on the fluorescence detection using the Softmax Pro Program (Molecular Devices).

### *In vitro S. mansoni* drug assay with microscopical readout

NTS from *S. mansoni* were obtained with the same vortex transformation procedure as mentioned above. The ice purification method was sufficient to separate the bodies from the tails. A lower antibiotic dose (1% penicillin-streptomycin mix) was used for the assays, as lower contamination occurred here. Evaluation was done microscopically using the same viability scale (0–3) as mentioned before.

### Statistical analysis

Arithmetic Means and standard deviation were calculated using Microsoft Excel® software for cercarial shedding patterns, transformation and purification factors, fluorescent signals and evaluated IC_50_ values. All values were tested for normality. Student’s one-sample *t*-test was used to analyze the statistical significance of differences between mean experimental and control values of the fluorescent values for each NTS drug assay. A P-value of < 0.05 was considered significant.

## Results

### Cercarial emergence rhythm

**Figure 1:**
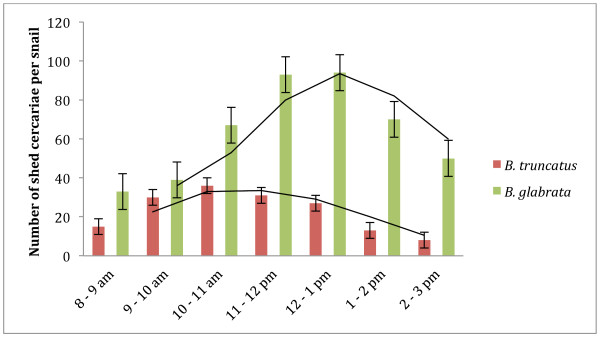
**Circadian rhythm of cercariae released from* S. haematobium * infected * B. truncatus *and* S. mansoni *infected* B. glabrata *snails.** Mean values of (n = 3) performed experiments are shown, bars indicate standard deviations.

The cercarial shedding of the two snail species *B. truncatus* and *B. glabrata* followed a clear circadian rhythm, after a simulated 12:12 hour light dark cycle in the laboratory. There was one shedding peak a day which slightly differed in time and intensity according to the species (Figure 1). The daily emergence pattern peak for *B. glabrata* occurred between 11 am and 1 pm, whereas for *B. truncatus* the peak occurred a little earlier between 10 am and 11.30 am. The total number of cercariae shed was 2.7-fold higher for *S. mansoni* infected *Biomphalaria* snails than for *S. haematobium* infected *Bulinus* snails.

### Transformation and purification

The highest transformation rate (mean of 69%) was observed for the vortex transformation. The previously described protocol for the vortex transformation [11] had to be slightly modified, such as increasing the number of pipetting (40x), increasing the vortex time (4 minutes) and using HBSS medium supplemented with 2% amphotericin B in order to achieve a continuously high transformation rate*.* Since high transformation rates were achieved with supplemented HBSS no other media were tested during the transformation procedure. A transformation rate of only 34% (mean value) was obtained with the chemical transformation. Both transformation methods were easy to perform.

In order to obtain the best method for purifying the NTS from the tails shed after transformation, a purification factor was calculated for three purification methods (Percoll®, ice and swirling method). The best purification factor was observed for Percoll® with 24.4 ± 11.4, followed by the swirling method with 11.7 ± 3.2 and the ice method presenting a mean purification factor of 3 ± 1.7.

### Optimal culture conditions for *S. haematobium* schistosomula

**Figure 2:**
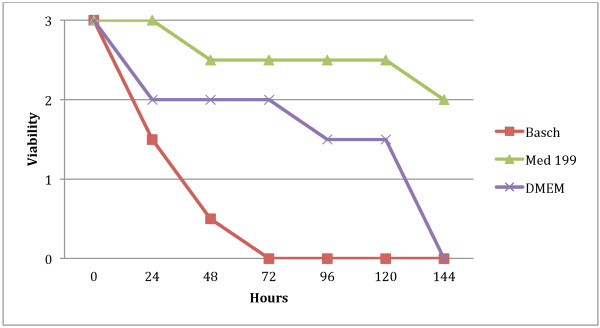
**Survival time of* S. haematobium * NTS (150–200 NTS per well) in different culture media**.

All media for *S. haematobium* were supplemented with 200 U/ml penicillin and 200 μg/ml streptomycin. This was the ideal concentration of antibiotics to get rid of bacterial contamination that occurred from *B. truncatus* snail excrement, which could not be eliminated during sedimentation in the first vortex transformation steps. Supplemented Medium 199 turned out to be the most suitable medium for the incubation of schistosomula obtained by transformation in supplemented HBSS. The parasites remained viable for 120 h with an average viability value of 2.5. On the other hand, all schistosomula had died by 72 hours in Basch medium and by 144 hours in DMEM (Figure 2).

### Evaluation of resazurin as potential fluorimetric marker for the *S. haematobium* NTS *in vitro* assay

**Figure 3:**
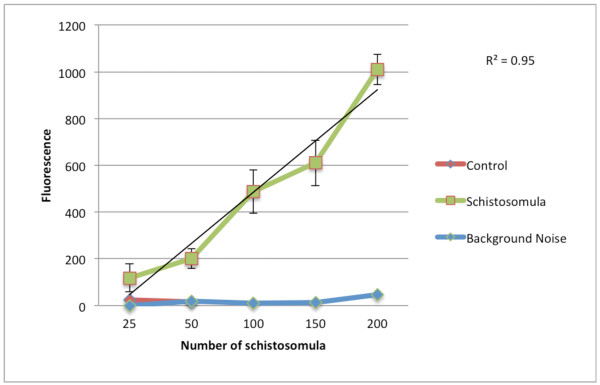
**Relationship between fluorescence signal and number of schistosomula (25–250 schistosomula per well) in the resazurin based assay.** Figure shows an average curve of four replicates, bars indicate standard deviations of the performed experiments. Fluorescence was measured after 24 hours of incubation. The fluorescence decreased with schistosomula concentrations above 200 per well. Linear regression is shown for data of 25-200 NTS.

In an initial experiment the relationship between the fluorescence signal of the converted resazurin and schistosomula numbers (0–250 NTS tested) was studied. A linear relationship (R² = 0.95) between fluorescence development and NTS number was observed within a range of 25–200 NTS per well, as depicted in Figure 3. No increase in the fluorescence signal was observed using 250 schistosomula per well and above. Dead schistosomula and medium did not show any significant background signals.

### *In vitro S. haematobium* NTS drug assay using microscopical readout compared with fluorimetric readout

As seen in Table 1, mefloquine showed the highest activity (IC_50_ value of 0.5 μM) followed by artesunate (1.4 μM), praziquantel (1.5 μM) and metrifonate (1.6 μM) against *S. haematobium* NTS using microscopical readout. For oxamniquine only low to moderate antischistosomal effects were observed against the schistosomular stage (IC_50_: 26.5 μM). Severe effects on the morphology of *S. haematobium* NTS were observed 24, 48 and 72 hours post exposure with praziquantel, mefloquine and artesunate. Concentrations of 90 μg/ml praziquantel and mefloquine killed the parasite within 48 and 24 hours, respectively. Both drugs caused strong morphological changes such as severe deformation and increased granularity. Metrifonate killed all the parasites, after 24 hours drug exposure with 90 μg/ml and after 72 hours at a concentration of 30 μg/ml. At lower concentrations such as 1.1 μg/ml, 3.3 μg/ml and 10 μg/ml, motility was reduced until hardly any movement could be observed after 48 and 72 hours. Oxamniquine treatment caused only slight morphological changes such as rounding but resulted in strongly reduced motility.

**Table 1:** **Comparison of the IC**_**50**_** values of tested drugs for* S. haematobium *and* S. mansoni *schistosomula at the 72 hours time point evaluated microscopically and for* S. haematobium * NTS additionally fluorimetrically with resazurin**

**Technique**	**Drug**	**IC**_**50**_**values (μM) (mean ± SD)**	
		***S. haematobium***	***S. mansoni***
Microscopy	Praziquantel	1.5 ± 1.1	0.7 ± 0.1
	Mefloquine	0.5 ± 0.2	0.7 ± 0.1
	Artesunate	1.4 ± 0.1	1.5 ± 0.6
	Metrifonate	1.6 ± 0.0	0.8 ± 0.1
	Oxamniquine	26.5 ± 3.4	11.8 ±0.0
Resazurin	Praziquantel	<3.5	ND
	Mefloquine	6.4	ND
	Artesunate	7.4	ND
	Metrifonate	348.3	ND
	Oxamniquine	>322.2	ND

IC_50_ values are calculated from three replicates at a concentration range of 1.1-90 μg/ml. ND: Not determined.

In a next step, IC_50_ values were determined in this assay using resazurin as viability marker. Praziquantel revealed the highest activity with an IC_50_ below 3.5 μM (P < 0.05) (Table 1). Mefloquine (6.4 μM) and artesunate (7.4 μM) showed similar promising activities whereas oxamniquine (>322.2 μM) and metrifonate (348.3 μM) were characterized by a low activity in this assay (all P < 0.05).

The IC_50_ values obtained for praziquantel, mefloquine and artesunate against *S. haematobium* NTS presented within similar ranges when evaluated microscopically or fluorimetrically. Oxamniquine lacked an antischistosomal effect according to the fluorimetric readout (IC_50_ > 322.2 μM) while a moderate activity was observed when the effects of the drug were assessed microscopically (26.5 μM). To note, great differences were also observed between the readouts for metrifonate (348.3 μM versus 1.6 μM).

### Comparison of IC_50_ values of *S. haematobium* and *S. mansoni* NTS

Using microscopy, very similar IC_50_ values of the 5 standard drugs were calculated for *S. haematobium* and *S. mansoni* NTS (Table 1).

## Discussion

*S. haematobium* is a neglected parasite that still affects several million people per annum [1-4]. There is currently one drug available as the core treatment of schistosomiasis and it is worrying that low cure rates with praziquantel have already been reported in several endemic countries for *S. mansoni*[18]. To our knowledge, we have for the first time established an *in vitro* drug sensitivity assay using *S. haematobium* NTS and additionally evaluated resazurin as a viability marker in this assay.

We first focused on the intermediate host snails and observed that the cercarial emergence rhythm of *B. glabrata* infected with *S. mansoni* as well as *B. truncatus* with *S. haematobium* showed one distinct peak for each species. It is important to state that these observations are based on long term laboratory isolates and the cercarial output of the imported *Bulinus* snails could also be influenced by the transportation between laboratories (~3 days of transport). However, the daily emergence pattern peak for *B. glabrata* occurred between 11 am and 1 pm, whereas for *B. truncatus* the peak occurred a little earlier between 10 am and 11.30 am. These results underline the fact that infected *B. glabrata* and *B. truncatus* snails show a distinct cercarial chronobiology [19,20]. It has been shown that *B. glabrata* shed approximately 2.7 times the number of cercariae per day (mean of 446 cercariae per snail) compared to *B. truncatus* (mean of 160 cercariae per snail), despite *B. truncatus* being exposed to a greater number of miracidia (10 per snail) than *B. glabrata* (8 per snail). We did not observe any influence of size or appearance of the snails such as calcareous deposit (calcification strips on the snail’s shell) on the daily cercarial shedding in *B. truncatus* snails. Surprisingly, these findings contradict a previous report [21] that had shown that cercarial output is related to the size of snail, meaning larger snails (9–10 mm diameter) shed much more cercariae than smaller sized snails (5–6 mm).

The mechanical transformation as described by Ramalho-Pinto *et al*. for *S. mansoni* was successfully tested on *S. haematobium* and yielded a transformation rate of 69% [11]. Overall both transformation methods, mechanical and chemical turned out to be very applicable on *S. haematobium* cercariae and were simple to perform. It is interesting to note that the chemical stimulation had a much lower transformation rate (30–41%) than the mechanical stimulation (59–87%). Salafsky *et al.*[22] reported that chemically transformed cercariae lost their osmoregulation ability at a much higher rate than during any other transformation method. Furthermore, Ramalho-Pinto *et al*. [11] observed that tail-less cercariae have increased water sensitivity as compared to fully intact cercariae. These findings might explain the reason for the lower transformation rate in chemical stimulation.

We made the observation that increasing the time of vortexing resulted in decreased fitness of the parasites over time (data not shown). For this reason, we did not further increase the vortex time. We also did not use the syringe method, which might be worthwhile testing in future studies to achieve even higher transformation rates.

After successfully transforming cercariae, schistosomula had to be purified from the tails shed and fully intact cercariae. Unfortunately, the ice method described by Fred Lewis [14] turned out to be less successful with *S. haematobium* and thus two other purification methods were tested, namely the swirling method and Percoll® method, which were previously described in the literature [13,14]. Both of these methods successfully separated tails from the schistosomula. Percoll® turned out to be twice as effective as the swirling method. The silica colloid gradient was non-toxic and did not affect any assay procedures [13]. Schistosomula isolated by centrifugation on Percoll® did not show any loss in viability, as evaluated by morphological examination. However, it must be emphasized that using Percoll® for the purification is an expensive method compared to the swirling method. Nevertheless, taking all advantages of the gradient together, Percoll® was used for all further transformation and purification experiments performed.

Drug sensitivity screenings were performed on newly transformed *S. haematobium* schistosomula, derived from successful vortex transformations, first with the classical microscopical readout. Note that, the assessment of the parasite viability microscopically *in vitro* is based on two parameters: regular or lack of movement of larvae (motility) and morphological changes such as granularity and shape alterations [22]. However, microscopic assessment is subjective, slow, labor intensive and requires experience [23,24]. For this reason, it is crucial to find other evaluation and screening methods based not only on microscopic examination. In our study the widely used viability indicator resazurin, the active component of Alamar Blue®, was evaluated.

In our preliminary studies it could be shown that a linear correlation exists between the fluorescence signal and viable transformed schistosomula. Fluorescence values per well increased proportionally to the number of schistosomula up to the amount of 200 schistosomula. The use of more than 200 schistosomula resulted in decreased fluorescence values. This might be due to crowding effects in the well.

A correlation is observed between microscopic and fluorometric readout treating *S. haematobium* with artesunate, praziquantel and mefloquine. All three drugs showed clear dose response relationships (decrease in viability with increasing concentrations) in both the microscopic and fluorometric readout. However, the determined IC_50_ values in the resazurin based assay presented a slightly higher range compared to values calculated based on microscopy. This indicates that the sensitivity of resazurin as an indicator of metabolic activity, shows a higher sensitivity on the detection of viability than the motility observations via the microscope. Furthermore, it is important to mention that in particular for the microscopic results for several of the tested drugs, the lowest concentrations tested were in the range of the calculated IC_50_ values (e.g. mefloquine), and lower concentrations were not tested in the present work resulting in extrapolated IC_50_ values. On the other hand, important morphological criteria are taken into account within the microscopic approach, which might be missed using fluorimetric viability markers. Interestingly, the fluorometric readout regarding oxamniquine and metrifonate treatment showed that NTS were still alive and metabolically functioning but the observation of the treated parasites via the microscope showed strong changes on motility and slight morphological changes of the treated schistosomula. Summarizing, one can state that a fluorimetric drug assay with *S. haematobium* NTS based on resazurin is efficient in detecting severely damaged respectively dead schistosomula, as already described for *S. mansoni* NTS [10]. Unfortunately, the assay cannot detect drug effects on the motility of the worms or drugs with slight morphological damages as demonstrated for oxamniquine and metrifonate.

In our panel of known antischistosomal drugs, metrifonate and oxamniquine were included though both drugs have been replaced by praziquantel and are not used any longer [6]. Interestingly, we observed a good *in vitro* activity of metrifonate, a drug recommended for treating infections with *S. haematobium*, on *S. mansoni* NTS, which is in line with a previous study using adult worms [25]. Oxamniquine (used for the treatment of *S. mansoni* infections) showed moderate activity against *S. haematobium* and *S. mansoni* NTS, a finding, which is in contrast to previous results using *in vitro* cultures with adult worms [26]. *S. haematobium* adults were not affected by oxamniquine, while a moderate activity was observed against *S. mansoni* adult worms [15,26]. These varying drug sensitivities between the schistosomular and adult stage might possibly be explained with differences in drug activating enzymes, the main target of oxamniquine [26].

## Conclusions

We have successfully developed an *in vitro* assay based on *S. haematobium* NTS. Using mechanical stress to transform cercariae into schistosomula instead of working with adult worms from mice and hamsters reduces the use of animals and thus stands in accordance with the 3Rs animal protection principles [9]. It should be highlighted that although schistosomula have great advantages when used for viability assays, it is crucial not to neglect adult schistosomes, as previously mentioned, differing susceptibilities on different parasite stages are not uncommon. Nonetheless schistosomula serve as an important starting point for the assessment of new drug candidates since it allows carrying out medium to high-throughput screenings.

The resazurin-based assay has proven to be effective, simple and possibly useful for large screening of drugs against *S. haematobium* NTS, though compounds acting solely on the motility or morphology of the worms (metrifonate, oxamniquine) might be missed.

However, it must be mentioned that working with *S. haematobium* schistosomula respectively, *B. truncatus* snails was more time consuming than working with *S. mansoni/ B. glabrata*. In addition to that, drug sensitivity screening for both species showed very similar results. Nevertheless, *S. haematobium* must not be neglected despite its more laborious work. *S. haematobium* infections still occur in most parts of Africa and are a major problem in developing countries [27]. For this reason, it is crucial to continue working with both species, possibly with *S. mansoni* first followed by testing of active compounds against *S. haematobium*.

## Competing interests

The authors declare that they have no competing interest.

## Authors’ contributions

MM, KI and JK designed the studies. MM carried out the experiments and wrote the first draft of the manuscript. KI and KJ revised the manuscript. All authors read and approved the final version of the manuscript.
